# Top 10 research priorities in colorectal cancer: results from the Colorectal Cancer Priority-Setting Partnership

**DOI:** 10.1007/s00432-022-04042-w

**Published:** 2022-05-17

**Authors:** Rosa Klotz, Magdalena Holze, Colette Dörr-Harim, Erich Grohmann, Barbara Nied, Burkhard Lebert, Susanne Weg-Remers, Claudia Lutz, Karin Meißler, Patrick Schloss, Charlotte Ullrich, Susanne Frankenhauser, Heidi Lutter, Diedrich Bühler, Azaz Ahmed, Toto Gronlund, André L. Mihaljevic, for the Forschungspartnerschaft Darmkrebs (www.forschungspartnerschaft.de), André Mihaljevic, Rosa Klotz, Colette Dörr-Harim, Steering Group Forschungspartnerschaft Darmkrebs/Priority Setting Colorectal Cancer, Diedrich Bühler, Colette Dörr-Harim, Susanne Frankenhauser, Sylvia Gapp-Frasch, Erich Grohmann, Volker Helmstädter, Christa Hey, Werner Hey, Rosa Klotz, André Mihaljevic, Burkhard Lebert, Claudia Lutz, Heidi Lutter, Claudia Lutz, Karin Meißler, Barbara Nied, Patrick Schloss, Susanne Stegmüller, Peter Stünzi, Angelika Trabold-Hauk, Charlotte Ullrich, Susanne Weg-Remers, Michel Wensing

**Affiliations:** 1 Department of General, Visceral and Transplantation Surgery, University Hospital Heidelberg, Heidelberg, Germany; 2 Study Centre of the German Society of Surgery, Heidelberg, Germany; 3 Department of General and Abdominal Surgery, University Hospital Ulm, Albert-Einstein-Allee 23, 89081 Ulm, Germany; 4 Department of Surgery (ulmCARES), Clinical Trial Centre, University Hospital Ulm, Ulm, Germany; 5 National Directorate, Deutsche ILCO e.V., Bonn, Germany; 6 Academy for Healthcare Professions Heidelberg, University Hospital Heidelberg, Heidelberg, Germany; 7 Cancer Information Center, German National Cancer Research Center (DKFZ), Heidelberg, Germany; 8 Frauenselbsthilfe Krebs Bundesverband e.V., Bonn, Germany; 9 Baden-Württemberg Section, Deutsche ILCO e.V., Stuttgart, Germany; 10 Department of General Medicine and Healthcare Research, University Hospital Heidelberg, Heidelberg, Germany; 11 Department of Interdisciplinary Emergency Rescue and Trauma Medicine, BG Hospital, Ludwigshafen, Germany; 12 Semi-Colon, Mönchengladbach, Germany; 13 National Association of Statutory Health Insurance Funds, Berlin, Germany; 14 National Center for Tumor Diseases, University Hospital Heidelberg, Heidelberg, Germany; 15 James Lind Alliance, National Institute for Health Research Evaluation, Trials and Studies Coordinating Centre, University of Southampton, Southampton, UK

**Keywords:** Patient participation, Priority-Setting Partnership, Forschungspartnerschaft, Colorectal neoplasms

## Abstract

**Background:**

Colorectal cancer (CRC) is the third most frequent cause of cancer death in the word. Which aspects of research into CRC should be accorded the highest priority remains unclear, because relevant stakeholders, such as patients, nurses, and physicians, played hardly any part in the development of research projects. The goal in forming the CRC Priority-Setting Partnership (PSP) was to bring all relevant stakeholders together to identify and prioritize unresolved research questions regarding the diagnosis, treatment, and follow-up of CRC.

**Methods:**

The CRC PSP worked in cooperation with the British James Lind Alliance. An initial nationwide survey was conducted, and evidence uncertainties were collected, categorized, summarized, and compared with available evidence from the literature. The as-yet unresolved questions were (provisionally) ranked in a second national wide survey, and at a concluding consensus workshop all stakeholders came together to finalize the rankings in a nominal group process and compile a top 10 list.

**Results:**

In the first survey (34% patients, 51% healthcare professionals, 15% unknown), 1102 submissions were made. After exclusion of duplicates and previously resolved questions, 66 topics were then ranked in the second survey (56% patients, 39% healthcare professionals, 5% unknown). This interim ranking process revealed distinct differences between relatives and healthcare professionals. The final top 10 list compiled at the consensus workshop covers a wide area of research topics.

**Conclusion:**

All relevant stakeholders in the CRC PSP worked together to identify and prioritize the top 10 evidence uncertainties. The results give researchers and funding bodies the opportunity to address the most patient-relevant research projects. It is the first detailed description of a PSP in Germany, and the first PSP on CRC care worldwide.

**Supplementary Information:**

The online version contains supplementary material available at 10.1007/s00432-022-04042-w.

## Introduction

Among the malignant diseases, colorectal carcinoma (CRC) is the third-ranked cause of death in Germany and worldwide (Zentrum für Krebsregisterdaten and GEKID 2021). The number of German residents ≥ 65 years is projected to increase from 16.8 million in 2010 to 23.7 million (+ 41%) in 2040 (Pritzkuleit and Katalinic 2016). Due to this demographic trend, the incidence of CRC will also rise sharply (Statistisches Bundesamt 2009; Motel-Klingebiel et al. 2010; Suzman and Beard 2011; Robert-Koch Institut and Gesellschaft der epidemiologischen Krebsregister in Deutschland e. V. 2015), probably growing from 79 to 120 per 100,000 inhabitants (Pritzkuleit and Katalinic 2016). CRC requires interdisciplinary and interprofessional care (cancer care continuum) (Feuerstein and Ganz 2011).

The topics of research projects are frequently determined by industry, e.g., pharmaceutical companies, or by public sponsors. Little is known about the perspectives of other relevant stakeholders, such as patients, family members, carers, nursing staff, treating physicians, or other health care professionals involved in care, because these groups generally do not become involved in the identification and prioritization of research topics. In many fields of medicine, this has led to discrepancies between the priorities of patients and the studies actually conducted (Crowe and Giles 2016).

Recent years have seen an increased awareness of the need to involve patients and the public in research (Jilani et al. 2020). Patient and public involvement should grant those concerned the opportunity to take an active part in the development of research programs and the design of research projects. An objective, transparent, and effective method of identifying and prioritizing evidence uncertainties is the concept of the “priority-setting partnership” (PSP, *Forschungspartnerschaft*) popularized by the James Lind Alliance (JLA, www.jla.nihr.ac.uk) (Partridge and Scadding 2004). The German PSP group (www.forschungspartnerschaft.de) recently published, in cooperation with the JLA, the first German PSP on the treatment of pancreatic cancer (Klotz et al. 2020). The goal of the research project presented here is to work together with patients, carers, nursing staff, treating physicians, and other relevant stakeholders as equal partners to identify and prioritize open questions regarding the diagnosis, treatment, and follow-up of CRC. It is the first detailed description of a PSP in Germany and the first PSP for CRC worldwide.

## Methods

The PSP was carried out in accordance with the published JLA guidelines (versions 9 and 10) (James Lind Alliance 2021). Every PSP consists of seven steps (Mihaljevic 2022).

### Step 1: Formation of a steering group and acquisition of partners

The project began with the formation of a steering group to supervise the entire process. The steering group was supported by an adviser from the JLA (TG). The members of the steering group defined the aims and scope of the CRC PSP (Supplement 1):

1. Transparent and systematic identification of unresolved research questions regarding the *diagnosis, treatment, and follow-up* of patients with CRC, taking account of the interests of all relevant stakeholders.
2. Joint prioritization of the research topics identified, taking account of the interests of all relevant stakeholders.
3. Publication und dissemination of the results.

CRC screening was explicitly excluded so as to restrict the PSP to manageable dimensions. The steering group met to discuss the progress of the PSP at regular intervals. All steps of the PSP were debated and agreed in the steering group. A core team (RK, CDH, MH, AM) implemented the steering group’s decisions. The steering group contacted a large number of self-help groups and professional societies to request their cooperation in the project, particularly with regard to the distribution of the two surveys.

### Step 2: First survey to identify unresolved research topics

The questions were formulated according to the recommendations of the JLA and included examples and prompts (Supplement 2). The questionnaire was distributed on paper as well as being made available on the homepages of the PSP and the partners in the project. It was also sent to CRC centers certified by the German Cancer Society *(Darmkrebszentren)* and to relevant professional associations. Moreover, patients and healthcare professionals were approached directly. The online questionnaire was implemented using the survey tool LimeSurvey. Given the anonymous nature of the questionnaire, the ethics committee of the University of Heidelberg determined that no legal consultation was required. The first questionnaire was available from 1 July 2020 to 31 October 2020.

### Step 3: Classification and formulation of the research questions

The members of the core team (RK, MH, CDH, AM) collected and processed all of the research questions proposed by the respondents to the first survey. Topics considered to be beyond the scope of the Colorectal Cancer PSP were referred to the steering group, where they were discussed and, if agreement was reached, excluded. Moreover, the individual topics were categorized according to content.

### Step 4: Review of available evidence and exclusion of questions already resolved

The members of the core team scrutinized CRC guidelines (as of October 2020) to identify further unresolved research questions and determine which of the questions raised by the survey respondents had already been answered (see list of guidelines in Supplement 7). The core team and steering group then refined the content and formulation of all remaining items so as to arrive at indicative questions in the PICO format (patient, intervention, comparison, outcome). In the course of this process, similar questions raised by several participants were amalgamated into one single question. Furthermore, questions that had already been resolved were eliminated from the pool of topics if the steering group was aware of the existence of evidence classified as level 1 a/b according to the Oxford Level of Evidence table (Oxford Centre for Evidence-Based Medicine 2009). The resulting catalog of indicative questions was compared with the available evidence as determined by means of a literature search. To this end, the Cochrane Database was searched by an information specialist and by the PSP core team. Again, a research topic was considered resolved if level1 a/b evidence was found (Oxford Centre for Evidence-Based Medicine 2009).

### Step 5: Second survey for the purpose of interim ranking

The catalog of indicative questions generated in step 4 served as the basis for the second survey. The questionnaire could be completed online or on paper at any time from 1 May 2021 to 31 July 2021. The participants were asked to select from the catalog the 10 research priorities they considered most important (top 10) (Supplement 3). In the digital version of the questionnaire, the topics were presented in a new random order each time the form was opened. As in the first survey, the respondents were asked to specify their sex, age group, and whether they were patients/carers or healthcare professionals.

### Step 6: Consensus workshop for final ranking

The final consensus workshop took place in Heidelberg on 11 September 2021. The JLA consultant (TG) ensured that the process adhered to the JLA guidelines (James Lind Alliance 2021). The workshop participants were patients/carers and healthcare professionals in equal numbers. The majority of the participants had not been involved in the PSP process prior to the workshop, but some were members of the steering group. Three specifically trained advisors (AA, CDH, RK) served as independent facilitators in the group discussions and ensured that the discussions were balanced. The research priorities were ranked according to the nominal group technique (James Lind Alliance 2021). The results of the interim prioritization were discussed and re-ranked in two rounds of discussion, in each of which three balanced small groups met in parallel. Based on the results of the second round of discussion, a concluding plenary session of all participants reached a consensus on the final top 10 list.

### Step 7: Publication and dissemination of results

After publication of the paper, results will be made available to patient organizations, funding bodies and researchers.

## Results

### Steering group and project partners

The balanced steering group of the Colorectal Cancer PSP had a total of 23 members. The group of 12 patients and carers included seven members of self-help organizations, while the 11 representatives of the healthcare professions came from nursing (outpatient, inpatient, oncological care, stoma care), various clinical specialties (general and colorectal surgery, gastroenterology, anesthesiology, palliative medicine, oncology), the Cancer Information Service of the German National Cancer Research Center (DKFZ), and the Department of General Medicine and Healthcare Research, University of Heidelberg. A representative of the National Association of Statutory Health Insurance Funds was also part of the healthcare professions group. The groups and organizations that supported the project by distributing the questionnaires and encouraging their members to take part are listed at www.forschungspartnerschaft.de/partnerschaften/darmkrebs/.

### First survey to identify unresolved research topics

**Fig. 1:**
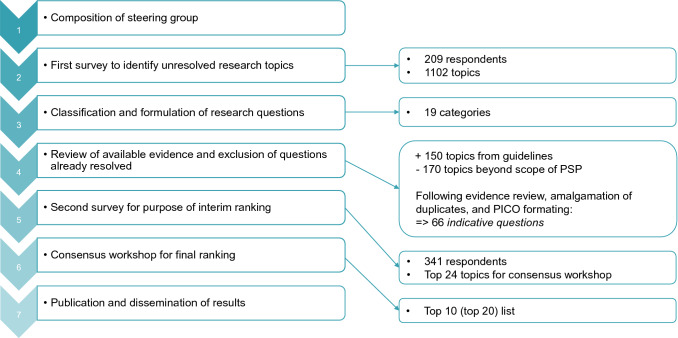
Flow chart of the Colorectal Cancer Priority-Setting Partnership

The first survey was completed by 209 participants (179 online, 30 on paper) (Fig. 1). A total of 1,102 research questions were proposed. Thirty-four percent of these topics (*n* = 373; 36% of the participants) were suggested by patients and carers, 51% (*n* = 558; 44% of the participants) came from healthcare professionals, and in 15% of cases (*n* = 171; 19% of the participants) it was not specified to which group the participant belonged. Details of the participants’ characteristics can be found in Table 1. Members of multiple medical disciplines took part in the survey, including surgery, internal medicine, anesthesiology, palliative medicine, psycho-oncology, and general medicine.

**Table 1:** Demographic parameters and roles of the respondents to the first and second surveys

	First survey *n* (%)	Second survey *n* (%)
Respondents	209 (100%)	341 (100%)
Patients/relatives	76 (36.4%)	192 (56.3%)
Healthcare professionals	93 (44.5%)	134 (39.3%)
Other/unknown	40 (19.1%)	15 (4.4%)
Age (patients only, *n* = 152)		
< 65 years	39 (51.3%)	93 (61.2%)
65–80 years	23 (30.3%)	50 (32.9%)
> 80 years	5 (6.6%)	2 (1.3%)
Unknown	9 (11.8%)	7 (4.6%)
Sex (patients only, *n* = 152)		
Male	28 (36.8%)	81 (53.3%)
Female	32 (42.1%)	64 (42.1%)
Unknown	16 (21.1%)	7 (4.6%)
Role (healthcare professionals only)		
Nurse	18 (19.4%)	41 (30.6%)
Stomatherapist	14 (15.1%)	1 (0.7%)
Internist	10 (10.8%)	10 (7.5%)
Surgeon	25 (26.9%)	37 (27.6%)
Radiologist	0 (0%)	0 (0%)
Psychotherapist	7 (7.5%)	4 (3.0%)
Anesthetist/Palliative medicine specialist	6 (6.5%)	7 (5.2%)
Other	8 (8.6%)	21 (15.7%)
Unknown	5 (5.4%)	13 (9.7%)

### Classification and formulation of the research questions

The proposed research topics were divided into the following 18 categories: (1) screening (*n* = 115); (2) diagnostic procedures (*n* = 20); (3) surgery (*n* = 40); (4) chemotherapy (*n* = 42); (5) radiotherapy (*n* = 26); (6) immunotherapy and individualized treatment (*n* = 25); (7) homeopathy (*n* = 56); (8) nutrition and digestion (*n* = 116); (9) stoma (*n* = 74); (10) quality of life (*n* = 21); (11) sport and exercise (*n* = 41); (12) psycho-oncology (*n* = 68); (13) pain therapy (*n* = 8); (14) palliative medicine (*n* = 9); (15) follow-up (*n* = 113); (16) pre-habilitation/re-habilitation (*n* = 23); (17) management and communication (*n* = 71); (18) other (*n* = 64). In agreement with the steering group, 170 of the 1,102 proposed topics were determined to be unrelated to the *diagnosis, treatment, and follow-up of CRC* and thus beyond the scope of the PSP. The remaining 932 topics were carried over to the following steps.

### Review of the available evidence and exclusion of questions already resolved

The core team identified 150 unresolved research questions in the CRC guidelines, and these were added to the 932 questions that had emerged from the first survey. As described above in the methods section, the steering group then distilled the unresolved topics into a catalog of indicative questions (*n* = 79), in the process amalgamating questions raised by several different participants into one single question. Following discussion with the steering group, a systematic search of the literature was carried out to exclude all topics for which level 1 a/b evidence already existed (*n* = 12). No topic was excluded without agreement being reached in the steering group. Two of the remaining 67 questions were amalgamated by the steering group, leaving 66 research topics for the second survey.

### Second survey for the purpose of interim ranking

The second survey (Supplement 3) for the purpose of interim ranking was completed by 341 participants (268 online, 73 on paper). Fifty-six percent of the participants were patients or carers, 39% were healthcare professionals, and the remaining 4% did not state to which of these two groups they belonged (Table 1). The results of interim ranking differed between the patients/ carers and the healthcare professionals (Supplement 4). Eight research questions were among the top 15 in both groups and were selected for the concluding workshop. The other seven topics from each group’s top 15 were added, giving a total of 22 questions. The steering group then debated the research questions ranked 16–20 in each group (patients/carers and healthcare professionals) to ensure that no topics assessed as relevant were excluded from the final round of consensus discussion. Two of these questions were deemed sufficiently important and added, yielding a list of 24 topics for the concluding workshop.

### Consensus workshop for final ranking

Twenty-six persons took part in the concluding workshop (*n* = 11 patients, *n* = 2 relations, *n* = 3 nurses, *n* = 9 physicians, *n* = 1 member of staff of the Cancer Information Service). The top 10 unresolved research priorities can be found in Table 2, and the topics ranked 11–20 are listed in Supplement 5 and 6 (German version). Four of the final top 10 priorities were in the original top 15 of both groups, two in the top 15 of the patients/carers only, and four in the top 15 of the healthcare professionals only.

**Table 2:** The top 10 unresolved research questions identified by the Colorectal Cancer Priority-Setting Partnership

1	How radical should the surgery be in the different stages of colorectal cancer, e.g., with regard to pelvic exenteration (= [radical] surgical removal of two or more pelvic organs), preservation of continence [ability to retain stool as desired], or lymph node excision?
2	What measures can be taken to help colorectal cancer patients cope with the disease and the adverse effects and consequences of treatment, e.g., bowel obstruction, diarrhea, anal inflammation, incontinence, parenteral nutrition (= nutrition via the veins), sexual problems, sequelae of stoma / stoma closure?
3	What potential is there for individualized treatment of patients with colorectal cancer, e.g., antibody therapies, targeted therapy with new drugs, or immunotherapy?
4	Does the involvement of specialized outpatient and inpatient personnel (nutritional counseling, oncology nurses, care service, stomatherapists, etc.) in the care of colorectal cancer patients improve the outcome?
5	What kind of specific preparation (pre-habilitation) has the potential to improve the outcome of the planned treatment (surgery, radiotherapy, chemotherapy, etc.) in colorectal cancer?
6	What role can be played by complementary medicine, e.g., meditation, osteopathy, traditional Chinese medicine, as a complement to conventional medicine (e.g., in regard to symptom relief and survival) in colorectal cancer?
7	In rectal cancer, how can LARS (low anterior resection syndrome = defecation problems after removal of the rectum) be effectively prevented (e.g., by reconstruction technique [= technique to restore the digestive tract, J pouch, transverse coloplasty, side-to-end anastomosis], pelvic neuromonitoring [= checking nerve function during the operation]) or treated?
8	How can the adverse effects of chemotherapy in colorectal cancer, e.g., polyneuropathy (= nerve damage associated with sensory disturbances and pain) or nausea, be avoided and treated?
9	What is the best sequence of treatment measures (chemotherapy, surgery, radiotherapy) for the different stages of colorectal cancer?
10	What measures have the potential to improve the quality of life and the general well-being of patients with colorectal cancer (e.g., nutritional counseling, psychosocial support)?

## Discussion

In a transparent, validated process, the Colorectal Cancer PSP identified the 10 most important research priorities (Table 2) from the total of over 1100 unresolved questions submitted. These top 10 questions, and even more so the extended top 20 priorities (Supplement 5), show the wide diversity of still unanswered questions. Moreover, the priorities of patients and relatives diverged from those of healthcare professionals. The PSP process accorded equal weight to the opinions of the patients/carers and other stakeholders. This project is the first PSP for colorectal cancer, and to our knowledge, only one other PSP has been performed in German-speaking countries (Klotz et al. 2020).

Patient and public partnerships are a relatively new development in medical research but have attained great significance (Richards et al. 2013). This trend has been described as an “ethical imperative and essential to improving the quality, safety, value, and sustainability of health systems and research” (British Medical Journal 2021). In Germany, too, public funding bodies are demanding increased involvement of patients in medical research (BMBF 2021). The lack of participation by patients is viewed as responsible for the waste of resources in biomedical research (Chalmers et al. 2014). The UK National Institute for Health Research differentiates between *involvement* (public involvement in research as research being carried out ‘with’ or ‘by’ members of the public rather than ‘to’, ‘about’ or ‘for’ them), *engagement* (when information and knowledge about research is provided and disseminated to patients or the public), and *participation* (when people take part in a research study) (NIHR 2021). Within involvement, one has to distinguish among (a) *consultation* (when patients or the public are asked their opinion and the views expressed are incorporated into the decision-making process), (b) *Collaboration* (“involves an ongoing partnership between (researches) and the members of the public…, where decisions about the research are shared”), and (c) *coproduction* (when the research is a joint project of scientists, patients, and the public in which responsibility and decision-making are shared from beginning to end) (NIHR 2021). The PSP concept presented here features elements of all three areas (involvement, engagement, participation) and represents coproduction of knowledge. It thus differs from other forms of patient involvement (e.g., patient advisory boards), whose function is frequently limited to consultation.

Priority-setting partnerships have become a common, internationally accepted way of determining research priorities, but PSPs on oncological topics are relatively rare (James Lind Alliance 2021a, 2021b). Furthermore, contrary to other countries like the UK and Canada (James Lind Alliance 2021a, 2021b), PSPs (Forschungspartnerschaften) are still uncommon in Germany. To our knowledge only one previous PSP in Germany has been performed: for pancreatic cancer treatment by our group (Klotz et al. 2020). In terms of scope, number of participants, and number of topics proposed, the CRC PSP is comparable with some of the previous oncological PSPs (Nixon et al. 2020), while other oncological PSPs were much smaller (Lophatananon et al. 2011). The results of the CRC PSP show the need for research in many different areas and pertain to basic, translational, clinical, and health services research. Most of the topics prioritized will only be able to be resolved in the context of interdisciplinary and interprofessional research partnerships. The fact that several (pilot) trials are currently addressing topics raised by our PSP is an indication of the relevance of the results. An example is the research into total neoadjuvant treatment (TNT) in rectal cancer patients with the possibility of organ-preservation (e.g., NO-CUT trial NCT03565029).

The Colorectal Cancer PSP has several limitations. To begin with, the important subject of CRC screening and prevention was not included. This was decided by the steering group at an early stage to ensure that the size of the PSP remained manageable. Moreover, the anonymous nature of the survey precluded acquisition of more detailed data on the respondents. The participants could therefore be described only in general terms, and demographic data, such as social status and level of education, were not recorded. Whether any subgroups might not have been adequately represented cannot be stated with sufficient certainty. Therefore, no comparisons could be made between the respondents to the first and second surveys. Because the questionnaires were available only in German, selection bias cannot be ruled out. However, the large number of questions submitted, and the broad diversity of fields covered, together with the balanced participation of patients/carers and healthcare professionals, makes it likely that the results are representative. A further limitation is the focus on the German-speaking countries. Depending on the healthcare system, other countries may to some extent yield other findings. However, this limitation applies also to other PSPs (James Lind Alliance). Another limitation applying to all FP is the (lack of) balance between too specific and too general research questions. In the process of the FP (see “Methods” section) specific research questions, that are relevant to only a certain group of stakeholders, might be eliminated. On the other hand, by formulating indicative questions by consensus, some research questions might appear rather general. However, the process guarantees the selection of research questions relevant to all stakeholders. Furthermore, the objective of the FP is not to formulate a specific research question that can be answered by a single specific project, but rather to formulate research questions that need to be addressed by several studies, thereby reflecting the complexity of CRC care and research. Finally, during the process of the PSP, evidence uncertainties are checked against existing guidelines, current at that time. As the JLA standards for excluding questions based on evidence criteria are high (level 1 a/b according to the Oxford; see “Methods” section), some research questions might have been carried forward in the PSP, contrary to guideline recommendations that are based on lower than level 1 a/b evidence.

In summary, the Colorectal Cancer Priority-Setting Partnership has identified and ranked evidence uncertainties in the diagnosis, treatment, and follow-up of CRC important to patients, family members, carers, physicians, and other stakeholders alike. This PSP give researchers and research funders the opportunity to focus their efforts on topics prioritized by all relevant stakeholders. It is the first detailed description of a PSP in Germany, and the first PSP on CRC care worldwide.

## Supplementary Information

Below is the link to the electronic supplementary material.

Supplement 1. The protocol of the Colorectal Cancer Priority-Setting Partnership

Supplement 2. Print version of the first questionnaire

Supplement 3. Print version of the second questionnaire

Supplement 4. Results of interim ranking

Supplement 5. Research priorities 11–20 of the Colorectal Cancer Priority-Setting Partnership

Supplement 6. Top 1–20 research priorities in German

Supplement 7. List of the guidelines screened
