# Supplementary material for: Top 10 research priorities in colorectal cancer: results from the Colorectal Cancer Priority-Setting Partnership
Source: J Cancer Res Clin Oncol. 2022 May 17;149(4):1561–8. doi: 10.1007/s00432-022-04042-w (PMC10020251; doi:10.1007/s00432-022-04042-w)
Supplement: Supplementary file 1 — Supplement 1. The protocol of the Colorectal Cancer Priority-Setting Partnership [file 432_2022_4042_MOESM1_ESM.pdf]

GEFÖRDERT VOM

Forschungspartnerschaft Darmkrebs
(englisch: "Priority Setting Partnership Colorectal Cancer")

PROTOKOLL

1. Ziel der PSP und Hintergrund

Der Zweck dieses Protokolls ist es, die Ziele und Verpflichtungen der Forschungspartnerschaft Darmkrebs (Priority Setting Partnership Colorectal Cancer) und die grundlegenden Rollen und Verantwortlichkeiten der beteiligten Partner festzulegen. Das Protokoll soll regelmäßig, mindestens jedoch halbjährlich, durch die Steering Group überprüft werden.

Die James Lind Alliance (JLA) ist eine gemeinnützige Initiative, die 2004 gegründet wurde. Sie bringt Patienten, Angehörige, Pflegende und Mitarbeiter des Gesundheitssystems (health care professionals) in einer *Priority Setting Partnership* (PSP, Forschungspartnerschaft) zusammen. Diese Partnerschaften identifizieren und priorisieren die wichtigsten Unklarheiten oder „unbeantworteten Fragen" zu Behandlungseffekten. Dies soll dabei helfen, sicherzustellen, dass sich die Förderer der Gesundheitsforschung dessen bewusst sind, was für Patienten[1], Angehörige, Pflegende und Mitarbeiter des Gesundheitssystems wirklich von Bedeutung ist.

Das kolorektale Karzinom (KRK, Darmkrebs) ist in Deutschland die zweithäufigste Krebsneuerkrankung bei Frauen und die dritthäufigste bei Männern, wobei das Alter der Erstdiagnose durchschnittlich über 70 Jahren liegt. Als Krebstodesursache in Deutschland steht das KRK in Deutschland an dritter Stelle. Aufgrund des demographischen Wandels in der Gesellschaft mit einer Zunahme der über 65-Jährigen von 16,8 Millionen im Jahre 2010 auf 23,7 Millionen im (+41,1%) im Jahre 2040, wird das KRK deutlich zunehmen. So wird die Inzidenz des KRK von 79,3 im Jahre 2010 auf 120,3 pro 100.000 Einwohner im Jahr 2040 steigen. Zusätzlich werden aufgrund der verbesserten Therapiemöglichkeiten mehr ältere Menschen nach einem KRK mit den möglichen Folgen der Erkrankung und Therapie leben.
Das KRK bedarf einer interdisziplinären, interprofessionellen und sektorenübergreifenden Versorgung (Cancer Care Continuum), welche von Diagnose, Therapie, Nachsorge und Palliation bis zur Sterbebegleitung reicht. Bei älteren Patienten mit KRK bestehen dabei aufgrund von Komorbiditäten, möglicher Gebrechlichkeit und vorbestehenden Einschränkungen im Alltag besondere Bedürfnisse, die in der aktuellen Versorgung nur ungenügend Berücksichtigung finden. Welche Fragen in der Behandlung von älteren Patienten mit KRK besonders dringlich adressiert und beforscht werden sollten, bleibt jedoch unklar, da betroffene Patienten, Angehörige, Patientenvertreter, Behandler (Ärzte, Pflegepersonal) sowie Leistungserbringer (Krankenkassen) in der Formulierung, Auswahl, Design und Durchführung von Forschungsvorhaben zum KRK ungenügend berücksichtigt werden. Zudem sind ältere Patienten mit KRK systematisch in klinischen Studien unterrepräsentiert. Als Folge werden ältere Patienten mit KRK auch im Zeitalter multidisziplinärer Tumorboards häufig über- oder untertherapiert.
Die Einbindung von Patienten mit KRK und ihren Angehörigen in die klinische Forschung ist bisher begrenzt. Solange diese Gruppen nicht an der Planung, Durchführung und Auswertung von klinischen Studien beteiligt sind, bleiben Defizite in der Therapie von älteren Patienten erhalten. Durch eine Priorisierung von Forschungsfragen unter Einbeziehung aller betroffenen Gruppen könnte dies in Zukunft vermieden werden.

[1] Der besseren Lesbarkeit wegen wird in diesem Dokument jeweils die männliche Form aufgeführt, meint jedoch beide Geschlechter.

2. Zweck und Ziele der Forschungspartnerschaft Darmkrebs

Im Rahmen der Forschungspartnerschaft Darmkrebs (Priority Setting Partnership Colorectal Cancer) sollen für Patienten ≥ 65 Jahre die wichtigsten unbeantworteten Forschungsfragen zur Diagnose, Therapie und Nachsorge des KRK identifiziert und priorisiert werden. Hierzu werden all jene einbezogen, die als Patienten, Angehörige, Patientenvertreter oder medizinisches Fachpersonal (Ärzte, Pflegende) vom KRK betroffen oder als Kostenträger direkt an der Versorgung dieser Patienten beteiligt sind. Da traditionell Forschungsthemen und -schwerpunkte von Wissenschaftlern oder Ärzten bestimmt werden, mit allenfalls geringer Beteiligung anderer relevanter Interessengruppen, blieb es bislang unklar, welche Forschungsfragen unter Berücksichtigung aller Interessengruppen am dringlichsten wissenschaftlich bearbeitet werden sollten. Die Ergebnisse des Projekts sollen daher zukünftig dazu dienen, gezielt patientenrelevante Forschungsvorhaben zu initiieren.

Folgende drei wissenschaftliche Arbeitsziele werden für das Projekt definiert:

1.) die transparente und systematische Identifikation von unbeantworteten Forschungsfragen zur Diagnose, Therapie und Nachsorge von Patienten ≥ 65 Jahre mit einem KRK unter Einbeziehung aller relevanter Interessengruppen.

2.) die gemeinsame Priorisierung dieser identifizierten Forschungsfragen unter Einbeziehung der relevanten Interessengruppen.

3.) die Veröffentlichung und Bekanntmachung der priorisierten Resultate auch gegenüber potenziellen Forschungsförderern, um gezielt die Diagnose, Therapie und Nachsorge von Patienten ≥ 65 Jahre mit einem KRK durch zukünftige Forschung, klinische Studien und Versorgungsprojekte zu verbessern.

3. Das “Steering Committee” (SC)

Die Forschungspartnerschaft Darmkrebs (Priority Setting Partnership Colorectal Cancer) wird von den folgenden Personen geführt und geleitet:

Leitung Prof. Dr. André L. Mihaljevic, MSc
(Chirurg, Klinik für Allgemein-, Viszeral- und Transplantationschirurgie Heidelberg)

Patientenvertreter und Angehörige:

- Sylvia Gapp-Frasch
- Erich Grohmann
- Christa Hey
- Werner Hey
- Burkhard Lebert
- Heidi Lutter
- Karin Meißler
- Barbara Nied
- Rudolph Ratheiser
- Prof. Dr. Patrick Schloss
- Susanne Stegmüller

- Peter Stünzi
- Angelika Trabold-Hauk

Mitarbeiter des Gesundheitssystems / Ärzte und Pfleger:

- Dr. Anne Berger, Oberärztin, Nationales Centrum für Tumorerkrankungen, Heidelberg
- Colette Dörr-Harim, Ärztin, Klinik für Allgemein-, Viszeral- und Transplantationschirurgie Heidelberg
- Dr. Susanne Frankenhauser, MME, Centrum für interdisziplinäre Rettungs- und Notfallmedizin, BG Ludwigshafen
- PD Dr. Volker Helmstädter, niedergelassener Arzt, Heidelberg
- Dr. Rosa Klotz, Ärztin, Klinik für Allgemein-, Viszeral- und Transplantationschirurgie Heidelberg
- Claudia Lutz, Fachkrankenschwester für Onkologie, Pflegeexpertin Stoma Kontinenz Wunde Klinik für Allgemein-, Viszeral- und Transplantationschirurgie Heidelberg

Methodische Berater, die nicht an der Priorisierungsaufgabe teilnehmen (siehe Kommentar 2):

- Dr. Charlotte Ullrich, Dipl. Soz. Wiss, Wissenschaftlerin, Allgemeinmedizin und Versorgungsforschung Heidelberg
- Prof. Dr. Michel Wensing, MSc, Wissenschaftler, Allgemeinmedizin und Versorgungsforschung Heidelberg

Andere Mitarbeiter des Gesundheitssystems

- Dr. Susanne Weg-Remers, Krebsinformationsdienst des DKFZ, Heidelberg
- Dr. Diedrich Bühler, GKV – Spitzenverband, Berlin

Weitere Mitarbeiter, die das Steering Committee unterstützen:

- Dr. Matthias Ganzinger (Informationsspezialist), Institut für Medizinische Biometrie und Informatik, Heidelberg
- Dr. Eva Kalkum (Systematische Literaturrecherche), Studienzentrum der Deutschen Gesellschaft für Chirurgie SDGC

Koordination:

- Dr. Alexandra Kunz, Studienzentrum der Deutschen Gesellschaft für Chirurgie SDGC
- Colette Dörr-Harim, Studienzentrum der Deutschen Gesellschaft für Chirurgie SDGC

Die Partnerschaft und der Priorisierungsprozess werden unterstützt durch

- Die James Lind Alliance (JLA) (Advisor: Toto Gronlund).

Im SC sind Repräsentanten von Patienten- und Angehörigengruppen und Mitarbeiter im Gesundheitssystem (Ärzte, Pflegende u.a.)[2], sowie klinische Wissenschaftler vertreten. Durch die Mitglieder

[2] In manchen Fällen wurde vorgeschlagen, dass Wissenschaftler auf dieser Stufe vertreten sind, um bezüglich der Form der Forschungsfragen zu beraten. Wissenschaftler dürfen jedoch an der Priorisierungsaufgabe nicht teilnehmen, um sicherzustellen, dass die finalen priorisierten Forschungsfragen diejenigen sind, die allein von Patienten, Pflegenden und Klinikern in Übereinstimmung mit dem Auftrag der JLA vereinbart wurden.

des SC werden die (zeitlichen) Ressourcen und die Expertise definiert, welche sie bei den einzelnen Schritten des Prozesses einbringen können. Die JLA wird das SC in seiner Arbeit beraten.

4. Weitere Partner

Es werden sowohl von Industrie und Pharmaindustrie unabhängige Organisationen als auch Einzelpersonen eingeladen, sich als Partner in die PSP einzubringen. Partner sind Gruppen oder Einzelpersonen, die sich verpflichten, die PSP zu unterstützen, indem sie den PSP Fragebogen verteilen und der PSP helfen, Fragen und Unklarheiten mit praktischer klinischer Bedeutung zu sammeln, die sich auf Diagnose, Therapie/Behandlung und Nachsorge des kolorektalen Karzinoms beziehen. Die Partner repräsentieren folgende Gruppen:

- Menschen ≥65 Jahre, die ein KRK haben oder hatten
- Pflegende/Betreuende/Vertreter von Menschen, die ein KRK haben oder hatten
- Ärzte, Krankenschwestern und medizinisches Fachpersonal mit klinischer Erfahrung im Bereich des KRK

Es ist wichtig, dass alle Organisationen, die diese Zielgruppen erreichen und sich für sie einsetzen, dazu eingeladen werden, sich an der PSP zu beteiligen. Der JLA Berater und das SC übernehmen die Verantwortung dafür, dass die verschiedenen am Projekt beteiligten Gruppen (Stakeholdergruppen) gleichermaßen zum Prozess beitragen können.

Ausschlusskriterien
Möglicherweise urteilt die JLA oder das SC über manche Organisationen (z.B. Pharmaindustrie), dass diese Interessenkonflikte haben, die mit dem Ziel der PSP kollidieren. Dies kann zu einer inakzeptablen Verzerrung der PSP führen. Da dies wahrscheinlich die endgültigen Ergebnisse der PSP beeinflussen würde, werden diese Organisationen nicht zur Teilnahme eingeladen. Es ist jedoch möglich, dass interessierte Parteien in einer rein beobachtenden Funktion teilnehmen, sofern das Steering Committee dies für hilfreich hält.

5. Methoden der PSP

Dieser Abschnitt beschreibt die Methoden, mit welchen die PSP ihre Ziele erreichen will. Der Prozess ist abhängig von der aktiven Beteiligung und dem Beitrag verschiedener Gruppen. Die angewendeten Methoden werden in Rücksprache zwischen den Mitgliedern des SC entsprechend ihren Zielen und Endpunkten festgelegt.

Schritt 1: Identifikation und Einladung potenzieller Partner

Potenzielle Partnerorganisationen werden durch Bekanntschaft und Kontaktaufnahme und über die Netzwerke der Mitglieder des SC identifiziert. Potenzielle Partner werden kontaktiert und über die Einrichtung und die Ziele der PSP PageCRC informiert.

Schritt 2: Initiales Stakeholdertreffen/Bewusstseinsbildung

Das initiale Stakeholdertreffen hat die folgenden Hauptziele:

- die potentiellen Mitglieder der PSP PageCRC zu begrüßen und vorzustellen

- den beabsichtigten Plan für die PSP PageCRC zu präsentieren
- eine Diskussion anzuregen, Fragen zu beantworten und Anliegen anzusprechen
- diejenigen potenziellen Partnerorganisationen, die sich der PSP verpflichten, und die Personen zu identifizieren, die diese Organisationen repräsentieren und als Hauptansprechpartner für die PSP dienen
- Grundsätze zu etablieren, auf Basis derer ein offener und transparenter Mechanismus aufgebaut werden kann, der zur Arbeit und dem Fortschritt der PSP beiträgt.

Schritt 3: Identifikation von Behandlungsunklarheiten

Jeder Partner bestimmt eine Methode, um von seinen Mitgliedern Fragen und Unklarheiten mit klinischer Bedeutung bezogen auf Diagnose, Therapie/Therapie und Nachsorge des KRKs zu akquirieren. Für diese Aufgabe stehen bis zu drei Monate zur Verfügung.

Die Methoden können entsprechend den Gegebenheiten und der Zugehörigkeit jeder Organisation gestaltet werden, müssen aber so transparent, offen und repräsentativ wie möglich sein. Die Methoden können Mitgliedertreffen, Beratungen, Fragebögen per Post, E-Mail oder webbasiert sein und Internetforen und Fokusgruppenarbeiten beinhalten.

Es werden vorhandene Informationsquellen bezüglich Behandlungsunklarheiten für Patienten und Kliniker durchsucht. Dies kann einen „Frage-Antwort-Service“ für Patienten, Pflegende und Kliniker beinhalten, sowie Forschungsempfehlungen in systematischen Übersichtsarbeiten und klinischen Leitlinien, Protokolle von geplanten systematischen Übersichtsarbeiten und Register laufender Forschung.

Der Startpunkt, um Quellen von Unklarheiten und Forschungsempfehlungen zu identifizierenden, ist die CENTRAL Bibliothek (http://www.cochranelibrary.com) und die MEDLINE Datenbank (z.B. via PubMed; www.ncbi.nlm.nih.gov/pubmed).

Schritt 4: Weiterentwicklung des Fragenkatalogs

Der Umfrageprozess wird „rohe“ unbeantwortete Fragen zu Behandlungseffekten ergeben. Diese „rohen“ Fragen werden von Prof. Dr. André Mihaljevic, Dr. Rosa Klotz, Colette Dörr-Harim, Magdalena Holze, Dr. Alexandra Kunz und Dr. Eva Kalkum zusammengestellt, kategorisiert und zu „zusammengestellten Fragen“ weiterentwickelt, die klar durch Forschung adressierbar und für alle verständlich sind. Ähnliche oder doppelte Fragen werden gegebenenfalls kombiniert/entfernt.

Systematische Übersichtsarbeiten, Leitlinien und medizinische Datenbanken werden durchsucht und es wird geprüft, in welchem Ausmaß diese „zusammengestellten Fragen“ durch die Forschung bereits beantwortet wurden. Manchmal werden Unklarheiten geäußert, die durch bestehende Forschungsevidenz beantwortet werden können, d. h. dass es sich um „unerkanntes Wissen“ handelt und nicht um Unklarheiten. Wenn eine Frage mit existierenden Informationen beantwortet werden kann, diese jedoch nicht bekannt ist, legt dies nahe, dass die Informationen denjenigen, die sie bräuchten, nicht effektiv genug kommuniziert wird. Entsprechend rät die JLA dringend, dass PSPs eine Aufzeichnung dieser zu beantwortenden Fragen machen und diese separat von den „wahren Unklarheiten“ zu behandeln, die während des PSP Prozesses untersucht werden.

Unklarheiten, die von früherer Forschung nicht ausreichend adressiert wurden, werden zusammengetragen und in einem Template erfasst. Dies wird die vorgenommene Kontrolle dokumentieren, mit der sichergestellt wird, dass die Unklarheiten noch nicht beantwortet wurden. Dies liegt in der Verantwortung des SC, die das Personal und die Ressourcen festlegen muss, um diese Verantwortlichkeit zu erbringen. Die Daten sollen

zur Veröffentlichung auf der Website an die JLA geschickt werden, wenn die PSP fertiggestellt ist, damit die Ergebnisse der PSP öffentlich verfügbar sind. Hierbei sollten Änderungen nachvollziehbar sein, die während des finalen Workshops gemacht wurden.

Die JLA wird an diesem Prozess als Beobachter teilnehmen und die Transparenz sicherstellen.

Schritt 5: Priorisierung – Interims- und finale Phase

Das Ziel der finalen Phase des Priority Setting Prozesses ist es, die identifizierten Unklarheiten zur Therapie oder zum Management des kolorektalen Karzinoms über einen Konsens zu priorisieren. Dies wird von den Mitgliedern des SC und weiteren Partnern, die Patienten und Kliniker repräsentieren, durchgeführt.

Die Zwischenstufe, um von einer „Longlist" von Unklarheiten zu einer „Shortlist" zu kommen, die im letzten Prioritätensetzungsworkshop (i.R. bis zu 30 Fragestellungen) erörtert werden soll, kann online durchgeführt werden, wobei Organisationen ihre Mitglieder konsultieren und ihre zehn wichtigsten Unklarheiten auswählen und bewerten. Dieser Schritt kann jedoch auch von Einzelpersonen absolviert werden.

Der letzte Schritt, um z. B. zehn priorisierte Unklarheiten zu erreichen, wird in einem persönlichen Gespräch mit Gruppendiskussionen und Plenarsitzungen durchgeführt.

Die Methoden für diesen Priorisierungsprozess werden in Abstimmung mit den Partnerorganisationen und mit der Beratung durch den JLA-Berater festgelegt. Zu den Methoden, die als potenziell nützlich in diesem Prozess identifiziert wurden, gehören: adaptierte Delphi-Techniken, Expertengremien oder nominale Gruppentechniken, Konsensusentwicklungskonferenz, elektronische Nominalgruppe und Online-Voting, interaktive Festsetzung der Forschungsagenda und Fokusgruppen.

Die JLA wird diesen Prozess unterstützen und Transparenz, Verantwortlichkeit und Fairness gewährleisten. Von den Teilnehmern wird erwartet, dass sie mögliche Interessenskonflikte im Voraus bekannt geben.

6. Verbreitung der Ergebnisse und Forschung

Ergebnisse und Forschung

Es wird erwartet, dass die Ergebnisse des PSP PageCRC an Finanzierungs- und Forschungsorganisationen wie die Deutsche Forschungsgemeinschaft (DGF), das Bundesministerium für Bildung und Forschung (BMBF) und das Bundesministerium für Gesundheit (BMG) berichtet werden. Von den forschenden Mitgliedern des SC und der Partnerorganisation wird erwartet, dass sie aus den priorisierten Unklarheiten Forschungsprojekte entwickeln, um die offenen Fragen zu beantworten.

Öffentlichkeitsarbeit

Neben der Information an Förderer, werden Partner und Mitglieder des SC darin unterstützt, die Ergebnisse der PSP PageCRC mittels interner und externer Kommunikationswege zu veröffentlichen. Das SC kann die Ergebnisse durch Berichte über den Prozess selbst in einfacher Sprache publizieren. Außerdem werden die Ergebnisse des PSPs vom Projektteam und dem SC als wissenschaftlicher Artikel publiziert. Allerdings sollte die Produktion einer wissenschaftlichen Publikation keinen Vorrang gegenüber der Veröffentlichung der endgültigen Ergebnisse haben.
