# Supplementary material for: Top 10 research priorities in colorectal cancer: results from the Colorectal Cancer Priority-Setting Partnership
Source: J Cancer Res Clin Oncol. 2022 May 17;149(4):1561–8. doi: 10.1007/s00432-022-04042-w (PMC10020251; doi:10.1007/s00432-022-04042-w)
Supplement: Supplementary file 2 — Supplement 2. Print version of the first questionnaire [file 432_2022_4042_MOESM2_ESM.pdf]

GEFÖRDERT VOM
Bundesministerium für Bildung und Forschung

Forschungsfragen zu Diagnose, Therapie und Nachsorge des Darmkrebses (kolorektales Karzinom) bei älteren Patienten

Wir sind eine Gruppe von Patienten, Angehörigen, Pflegenden und Ärzten, die sich zusammengeschlossen haben, um die wichtigsten unbeantworteten Forschungsfragen zur Diagnose, Therapie und Nachsorge des Darmkrebses zu ermitteln.

Wir möchten Sie daher bitten, **Fragen zum Darmkrebs** zu nennen, die Ihrer Meinung nach in der Zukunft **durch die Forschung** beantwortet werden sollen.

- Bitte beantworten Sie die Fragen aus **Ihrer eigenen Erfahrung** heraus.
- **Sie müssen nicht alle Fragen beantworten** und können so viel oder so wenig schreiben, wie Sie möchten.
- Alle Antworten werden **anonym** bearbeitet.

Wichtig
Bei der Umfrage werden unbeantwortete Fragen gesucht, die zukünftig durch die Forschung bearbeitet werden sollen.

Beispiel
Sie hatten als Patient/in nach einer Darmkrebsoperation langfristig Verdauungsstörungen.

➔ Als unbeantwortete Forschungsfrage für unser Projekt könnten Sie daher z.B. die Frage formulieren: „Welche Ernährung wird nach Operationen beim Darmkrebs am besten vertragen?“

☐ Ich möchte an der Umfrage teilnehmen

1. Aus Ihrer Erfahrung mit Darmkrebs, welche Fragen sollten zur Diagnose (Feststellung und Untersuchung) der Erkrankung durch die Forschung beantwortet werden?

2. Aus Ihrer Erfahrung mit Darmkrebs, welche Fragen sollten zur Therapie der Erkrankung durch die Forschung beantwortet werden? Zur Behandlung gehören z.B. Chirurgie/Operation, medikamentöse Tumorbehandlung (z.B. Chemotherapie), Bestrahlungstherapie, Schmerz- und Palliativbehandlung.

3. Aus Ihrer Erfahrung mit Darmkrebs, welche Fragen sollten zur unterstützenden und ergänzenden Therapie der Erkrankung durch die Forschung beantwortet werden? Zur unterstützenden und ergänzenden Behandlung gehören z. B. Stomaversorgung, pflegerische Versorgung, Naturheilverfahren, Komplementärmedizin.

4. Aus Ihrer Erfahrung mit Darmkrebs, welche Fragen sollten zum täglichen Leben mit der Erkrankung durch die Forschung beantwortet werden? Dazu gehören z. B. Bewegung, Ernährung, Verdauung, psychosoziale Unterstützung, Rehabilitation.

5. Aus Ihrer Erfahrung mit Darmkrebs, welche Fragen sollten zur Nachsorge der Erkrankung durch die Forschung beantwortet werden?

6. Gibt es sonst noch etwas, was Sie uns für dieses Projekt mitteilen möchten?

Wir bitten Sie nun noch um weitere Informationen zu Ihrer Person:

Welche der folgenden Beschreibungen trifft am besten auf Sie zu (ggf. mehrere Angaben):

☐ Ich bin Patient/in mit Darmkrebs (kolorektales Karzinom)

☐ Ich bin Angehörige/r eines/r Patienten/in mit Darmkrebs (kolorektales Karzinom)

☐ Ich bin beruflich in der Versorgung von Patienten/innen mit Darmkrebs (kolorektales Karzinom) tätig,

☐ Anderes, nämlich:____

☐ Möchte ich nicht beantworten

<u>Für Patienten und Angehörige</u>

Wir hätten gerne folgende Informationen zur erkrankten Person:

1. Alter der erkrankten Person

   ☐ Unter 65 Jahre ☐ 65 bis 80 Jahre ☐ über 80 Jahre
   ☐ möchte ich nicht beantworten

2. Geschlecht der erkrankten Person

   ☐ weiblich ☐ männlich
   ☐ möchte ich nicht beantworten

3. In welchem Alter wurde der Darmkrebs bei der erkrankten Person festgestellt?

   ☐ Unter 65 Jahre ☐ 65 bis 80 Jahre ☐ über 80 Jahre
   ☐ möchte ich nicht beantworten

Wenn Sie beruflich in der Versorgung von älteren Patienten/innen mit der Krebserkrankung des Dick- und Mastdarms (kolorektales Karzinom) tätig sind:

Beruf:	
Ggf. Fachrichtung oder Zusatzbezeichnung angeben:	

Indem Sie an der Umfrage teilnehmen, willigen Sie ein, dass wir Ihre Fragen anonym veröffentlichen dürfen.

Sie können uns helfen, weitere Rückmeldungen auf unseren Fragebogen zu erhalten, wenn Sie den Link zur Online-Umfrage oder eine Kopie des Formulars an andere Personen weitergeben.

Wir bedanken uns für Ihre Teilnahme an diesem wichtigen Projekt!

Wenn Sie die Umfrage in Papierform beantworten, bitten wir Sie, die Antworten an folgende Adresse zu schicken (bitte anonym, also ohne Absender):

Postanschrift: Studienzentrum der Deutschen Gesellschaft für Chirurgie (SDGC)
Colette Dörr-Harim
Im Neuenheimer Feld 130.3
69120 Heidelberg

Fax: 06221-5633850
E-Mail: colette.doerr-harim@med.uni-heidelberg.de
