# Supplementary material for: Top 10 research priorities in colorectal cancer: results from the Colorectal Cancer Priority-Setting Partnership
Source: J Cancer Res Clin Oncol. 2022 May 17;149(4):1561–8. doi: 10.1007/s00432-022-04042-w (PMC10020251; doi:10.1007/s00432-022-04042-w)
Supplement: Supplementary file 3 — Supplement 3. Print version of the second questionnaire [file 432_2022_4042_MOESM3_ESM.pdf]

Priorisierung von Forschungsfragen Diagnose, Therapie und Nachsorge des Darmkrebses (kolorektales Karzinom) bei älteren Patienten

Im Projekt "Forschungspartnerschaft Darmkrebs" (PageCRC) arbeiten Patienten, Angehörige, Pflegende und Ärzte der verschiedensten Fachbereiche zusammen. Ziel ist es, die wichtigsten unbeantworteten Forschungsfragen zur Diagnose, Therapie und Nachsorge des Darmkrebses zu ermitteln und zu gewichten (priorisieren), damit diese in der Zukunft durch Forschungsprojekte beantwortet werden können.

Im ersten Projektteil haben wir in einer deutschlandweiten Umfrage offene Fragen gesammelt. Unsere Projektgruppe hat alle eingereichten Fragen aus dieser ersten Umfrage analysiert. Fragen, die das gleiche Thema betreffen, wurden zusammengefasst. Des Weiteren wurden alle bereits von der Forschung beantworteten Fragen entfernt. Das Ergebnis ist die nachfolgende Liste aus 66 Fragen.

Wenn sie persönlich betroffen sind, Sie also Patient oder Angehöriger sind oder Patienten mit Darmkrebs betreuen, bitten wir Sie um Ihre Teilnahme an dieser Umfrage:

Lesen Sie sich **alle Fragen in Ruhe** durch. **Wählen** Sie anschließend **bis zu 10 Forschungsfragen** aus der Liste von 66 Fragen aus, die Sie persönlich für **besonders wichtig** halten. Gehen Sie dabei von Ihrer eigenen Erfahrung aus. Wählen Sie also solche Fragen aus, die von der Forschung besonders dringlich (vorrangig) bearbeitet werden sollten.

Alle Antworten werden anonym bearbeitet.

Die Umfrage dauert ca. 10-15 Minuten.

□ Ich möchte an der Umfrage teilnehmen

Forschungsfragen

Bitte wählen Sie aus der untenstehenden Liste **diejenigen 10 Forschungsfragen aus, die Ihrer Meinung nach am dringlichsten von der Forschung beantwortet werden sollen.**

Nr.	X = wichtig	Frage
1		In welchen Altersgrenzen und Zeitintervallen bzw. bei welchen Beschwerden sollten je nach Patienteneigenschaften (z. B. familiäre Häufung, Vorerkrankungen) Screeningmaßnahmen auf Darmkrebs erfolgen?
2		Welche weniger invasiven Screeningmaßnahmen alternativ zur Darmspiegelung gibt es und wie effektiv sind diese (z.B. Bildgebung, Bluttests, Enddarmspiegelung, „Liquid biopsy“, Spiegelung mittels Videokapsel)?
3		Wie kann die Teilnahme an Früherkennungsmaßnahmen für Darmkrebs in der Bevölkerung erhöht werden?
4		Wie kann man bei Darmkrebs bei Diagnosestellung das Therapieansprechen und die individuelle Prognose vorhersagen (z. B. durch Tumormarker)?
5		Welchen Nutzen haben Patienten mit Darmkrebs durch eine Vorstellung im Tumorboard (interdisziplinäre Besprechung unter Einbeziehung von Ärzten verschiedener Fachrichtungen)?
6		Wie kann das Risiko reduziert werden, dass Tumore im Rahmen der Darmspiegelung übersehen werden?
7		Welche bildgebenden Verfahren (z. B. endoskopischer (= bei der Darmspieglung angewandter) Ultraschall, PET-/ Computertomografie) sind bei Darmkrebs im Rahmen des Stagings (= Stadienbestimmung) je nach Befund sinnvoll?
8		Bei welchen Darmkrebs-Befunden (z.B. bei Frühkarzinomen) ist eine endoskopische Intervention (= Therapie im Rahmen einer Darmspiegelung, also ohne Operation) ausreichend?
9		Welche intraoperativen (= während der Operation des Darmkrebses) Möglichkeiten gibt es, den Tumor bzw. sein Ausmaß zu erkennen?
10		Wann sollte bei Darmkrebs welcher Operationszugang gewählt werden (z. B. offen = großer Bauchschnitt/laparoskopisch = mittels Bauchspiegelung/robotisch = mittels Operationsroboter)?
11		Wie können Komplikationen (z. B. Nahtundichtigkeiten, Wundheilungsstörungen) nach der Operation des Darmkrebses vermieden, reduziert und behandelt werden?
12		Wie häufig sollte der Operateur/das Zentrum eine Darmkrebsoperation durchführen (Mindestoperationszahlen pro Jahr), um eine hohe Operationsqualität zu erreichen?
13		Bei welchen Darmkrebsoperationen ist eine Darmspülung/prophylaktische (vorbeugende) Antibiotikagabe vor der Operation sinnvoll?
14		Wie radikal muss bei Darmkrebs je nach Tumorstadium operiert werden (z.B. in Bezug auf Beckenexenteration (= (radikale) operative Entfernung von mehreren Organen im Becken), Kontinenzerhaltung (Fähigkeit, Stuhl willkürlich zurückzuhalten), Lymphknotenentfernung)?
15		In welchen Fällen (je nach Patienteneigenschaften, Tumorstadium, ggf. erfolgter neoadjuvanter (= vor der Operation angewandte) Therapie) ist eine adjuvante (= nach der Operation angewandte) Chemotherapie bei Darmkrebs sinnvoll?
16		Wie können bei Darmkrebs Nebenwirkungen der Chemotherapie (z.B. Polyneuropathie (= Schädigung von Nerven, die mit Missempfindungen und Schmerzen einhergeht), Übelkeit) vermieden und behandelt werden?

Nr.	X = wichtig	Frage
17		In welchen Fällen (z. B. je nach Patienteneigenschaften, Tumorstadium) profitieren Patienten mit Darmkrebs von einer neoadjuvanten (= vorbereitend auf die Operation angewandte) Chemotherapie?
18		Welches Chemotherapieregime (Therapieschema) ist bei Darmkrebs in welchem Tumorstadium unter Einbezug der Patienteneigenschaften am besten geeignet?
19		In welchem Zeitintervall nach einer Darmkrebs-Operation und wie lange sollte eine adjuvante (= nach der Operation angewandte) Chemotherapie erfolgen?
20		Wie beeinflusst Methadon die Chemotherapie bei Darmkrebs (z.B. in Bezug auf Ansprechen, Nebenwirkungen)?
21		In welchen Fällen (je nach Patienteneigenschaften, Tumorstadium) und in welchem Ausmaß ist eine neoadjuvante (= vor der Operation angewandte) Strahlentherapie bei Enddarmkrebs sinnvoll?
22		In welchen Fällen kann bei Enddarmkrebs nach neoadjuvanter (vor der Operation angewandter) Radiochemotherapie (Kombination aus Strahlen- und Chemotherapie) und kompletter Remission (vollständigem Ansprechen) auf eine Operation verzichtet werden?
23		Wie können bei Darmkrebs Nebenwirkungen und Folgen der Strahlentherapie vermieden und behandelt werden?
24		Hat die intraoperative (= während der Operation durchgeführte) Bestrahlung bei lokal (im Bereich des Ersttumors) fortgeschrittenem Darmkrebs einen Stellenwert?
25		Welche Möglichkeiten bietet die personalisierte Therapie (z. B. Antikörpergabe, „targeted therapy“ (= zielgerichtete Therapie mit neuartigen Medikamenten) oder eine Immuntherapie bei Darmkrebs?
26		Welchen Stellenwert hat die Komplementärmedizin (z. B. Homöopathie, Meditation, Naturheilverfahren, Osteopathie, traditionelle chinesische Medizin) ergänzend zur Schulmedizin (z. B. in Bezug auf Symptomlinderung und Überleben) bei Darmkrebs?
27		In welchen Fällen profitieren Patienten bei der Darmkrebsoperation von einer protektiven/temporären Stomaanlage und in welchen Fällen ist sie nicht erforderlich?
28		Ist mit einem Stoma auf Dauer ein Leben mit guter Lebensqualität möglich?
29		Welche Maßnahmen können das Leben nach Stomaanlage verbessern (z.B. in Bezug auf Ernährung, Komplikationen, psychische Situation, Verdauung, Versorgung)?
30		Wann ist bei Darmkrebs der beste Zeitpunkt für eine Stomarückverlagerung (z. B. vor der Chemotherapie)?
31		Kann bei Darmkrebs der Erfolg/das Risiko einer Stomarückverlagerung vor der Operation beurteilt und verbessert werden (Schließmuskel- und Beckenbodentraining)?
32		Wird bei Darmkrebs das Leben nach Stomaanlage durch eine Betreuung durch Stomatherapeuten verbessert und wie sollen die Rahmenbedingungen beschaffen sein (z.B. ambulant, stationär, vor der Operation, Dauer der Betreuung)?
33		Welche Maßnahmen helfen bei Darmkrebs bei der Bewältigung der Erkrankung und der Therapienebenwirkungen und -folgen (z. B. Darmverschluss, Durchfälle, Entzündungen am After, Inkontinenz, parenterale Ernährung (= Ernährung über die Venen), sexuelle Probleme, Folgen der Stomarückverlagerung)?
34		Welche Maßnahmen können die Lebensqualität und den Allgemeinzustand bei Darmkrebs verbessern (z.B. Ernährung, Gespräche, Kultur, psychosoziale Unterstützung)?

Nr.	X = wichtig	Frage
35		Welche spezifische Ernährung ist bei Darmkrebs (auch nach der Operation, ggf. mit Stomaanlage) sinnvoll?
36		Wie kann bei Darmkrebs der Ernährungsstatus verbessert werden (z.B. in Bezug auf Appetitlosigkeit, ergänzende parenterale (= über die Vene verabreichte) Ernährung, Zusatzstoffe)?
37		Welche Folgen haben bei Darmkrebs Operation und Strahlentherapie in Bezug auf die Kontinenz (= Fähigkeit, Stuhl willkürlich zurückzuhalten), das Mikrobiom (= Mikroorganismen des Darmes), die Resorption (= Aufnahme von Nahrungsbestandteilen) und die Verdauung und wie kann man diese reduzieren?
38		Was ist bei Darmkrebs die beste Therapie bei Verdauungsstörungen?
39		Wie kann bei Enddarmkrebs LARS (Low anterior resection syndrom = gestörte Defäkation nach Enddarmentfernung) effektiv verhindert (z. B. mittels Rekonstruktionstechnik (= Technik der Wiederherstellung des Verdauungsweges, J-Pouch, transverse Kolosplastie, Seit-zu-End-Anastomose), pelvines Neuromonitoring (= Kontrolle der Nervenfunktion während der Operation)) oder behandelt werden (z. B. Biofeedback (= Erfassung und Darstellung bestimmter körpereigener Signale), Fasten, Irrigation (= Spülung des Darmes))?
40		Welche Sport- und Bewegungsarten sind bei Darmkrebs in welcher Situation (z. B. nach der Operation ggf. mit Stomaanlage oder während der Chemotherapie) möglich, wie können sie das Outcome (Ergebnis) z. B. in Bezug auf Hernienprophylaxe (= Vorbeugung von Bauchwandbrüchen), Komplikationsrate und Rezidivrisiko beeinflussen und welche sollten vermieden werden?
41		Wie können Patienten mit Darmkrebs und deren Angehörige am besten psychoonkologisch und psychosozial unterstützt werden (z. B. mittels achtsamkeitsbasierter Stressreduktion, Biofeedback (= Erfassung und Darstellung bestimmter körpereigener Signale), Gesprächskreisen, Informationen, Massagen)?
42		Welche Effekte hat bei Darmkrebs die psychoonkologische und psychosoziale Betreuung (auch Meditation) auf Lebensqualität und Überleben?
43		Welche psychosozialen Faktoren (z.B. Humor, eigene Einstellung, Resilienz (= psychische Widerstandskraft), Selbstwirksamkeit) haben bei Darmkrebs Auswirkungen auf Lebensqualität und Outcome (= Ergebnis)?
44		Welchen Einfluss haben bei Darmkrebs die Art der Anästhesie (z.B. Art der Vollnarkose, Kombination mit verschiedenen Schmerzkathetern) und die Art und der Schmerztherapie nach der Operation auf das Outcome (= Ergebnis)?
45		Was ist die beste Schmerztherapie bei Darmkrebs und gibt es spezielle Schmerzkonzepte/Bewältigungsstrategie (Copingstrategien)?
46		Wann sollte bei Darmkrebs die Palliativmedizin involviert werden und welche Maßnahmen sollte sie beinhalten?
47		In welchen Intervallen und wie lange sollte je nach Tumorstadium und Risikoprofil die Nachsorge bei Darmkrebs auch über die Heilungsbewährungsfrist von 5 Jahren hinaus durchgeführt werden und welche Untersuchungen (z. B. auch neue Biomarker, "liquid biopsy" (= Nachweis von Tumorzellen aus Blut), "maßgeschneiderte Nachsorge") sollte sie beinhalten, auch für die Therapiefolgen?
48		Durch wen sollte bei Darmkrebs die Nachsorge durchgeführt werden (z.B. Hausarzt, Nachsorgezentren, Onkologe) und wie kann die Compliance (= aktive Mitwirkung der Patienten) verbessert werden (z. B. PROMs = patient reported outcomes measures =

Nr.	X = wichtig	Frage
		Instrumente zur Patientenselbstdokumentation des Behandlungsergebnisses, Apps, Nachsorgepass)?
49		Wie können bei Darmkrebs Lebensqualität und Rezidivrisiko während der Nachsorge beeinflusst werden (z.B. Ernährung, Sport, psychosoziale Faktoren)?
50		In welchem Setting (Rahmenbedingungen, z. B. ambulant/stationär, welcher Zeitpunkt nach der Operation/begleitend zur palliativen Chemotherapie) kann eine Rehabilitationsmaßnahme das Therapieansprechen und die Therapiefolgen bei Darmkrebs verbessern und was sollte diese beinhalten?
51		Welche gezielte Vorbereitung (Prehabilitation) kann das Outcome (= Ergebnis) der geplanten Therapie (Operation, Bestrahlung, Chemotherapie etc.) bei Darmkrebs verbessern?
52		Welches Fachpersonal (z.B. ambulanter Pflegedienst, Darmzentrum, Ernährungsberatung, onkologische Fachpflegekraft, Stomatherapeut) ist für die Betreuung von Darmkrebspatienten erforderlich und verbessert dies das Outcome (= Ergebnis)?
53		Wie kann bei Darmkrebs die interdisziplinäre und intersektorale Zusammenarbeit (= Zusammenarbeit zwischen den verschiedenen an der Therapie beteiligten Fachkräften) verbessert werden (z. B. digitale Möglichkeiten, klare Ansprechpartner)?
54		Wie kann bei Darmkrebs die Arzt-Patienten Kommunikation und Aufklärung der Patienten (z. B. bezüglich Folgen und Nebenwirkungen der Therapie, Therapieentscheidungen) verbessert werden (z. B. Farbfotos aus dem OP, Patienteninformationsmappe)?
55		Verbessern die Angebote von Selbsthilfegruppen, Selbsthilfeinitiativen und/oder Selbsthilfeverbänden die Lebensqualität, die Gesundheitskompetenz oder das Überleben bei Patienten mit Darmkrebs?
56		Was ist bei Darmkrebs die beste Reihenfolge der unterschiedlichen Therapiemöglichkeiten (Chemotherapie, Operation, Strahlentherapie) abhängig vom Tumorstadium?
57		Welchen Nutzen bringen bei Darmkrebs patient-reported outcome measures (= Instrumente zur Patientenselbstdokumentation des Behandlungsergebnisses)?
58		Welchen Nutzen haben Protokolle ähnlich den ERAS-Prinzipien (Behandlung, die zu einer schnellen Erholung nach der Operation führt), um Patienten mit Darmkrebs nach Chemo-, Strahlen- und Immuntherapien wieder fit zu machen?
59		Wie kann für den einzelnen Patienten mit Darmkrebs nach Abwägung der persönlichen Prioritäten (z.B. Lebensqualität, Überleben) die individuell beste Therapie festgelegt werden?
60		Was ist bei Darmkrebs abhängig vom Befund (z. B. bezüglich Tumorstadium, Zeitpunkt des Auftretens und Anzahl der Metastasierung) die beste Therapie von Lebermetastasen (z.B. Cyberknife (robotergestützter Linearbeschleuniger für Strahlenchirurgie), Irreversible Elektroporation (= Weichgewebeablationsverfahren), Mikrowellenablation (= Zerstörung des Tumors durch Hitze von innen), Chemotherapie, Operation, Radiofrequenzablation (= Zerstörung des Tumors durch Zuführung von Hitze mit Hochfrequenzstrom), TACE/transarterielle Chemoembolisation (= gezielte Verstopfung von Arterien durch Chemotherapie))?
61		Was ist bei Darmkrebs je nach Tumorstadium die beste Therapie von Lungenmetastasen (z. B. Operation, stereotaktische Ablation = Abtragung von Gewebe mittels Hochpräzisionsbestrahlung)?

Nr.	X = wichtig	Frage
62		Sollten Patienten mit Darmkrebs täglich Aspirin einnehmen?
63		Welche Diagnostik soll bei Darmkrebs am OP-Präparat erfolgen und welche Informationen soll der pathologische Bericht beinhalten?
64		Ist bei Darmkrebs die Sedierung (Beruhigung) mit Propofol (= Schlaf-/Narkosemittel) im Rahmen der ambulanten Darmspiegelung durch den behandelnden Gastroenterologen sicher?
65		Was ist bei Darmkrebs die beste Therapie bei Peritonealkarzinose (= Tumorstreuung ins Bauchfell)?
66		Sollte bei Darmkrebs bei nicht-resektablen Metastasen (= Tumorstreuung, die nicht vollständig operativ entfernt werden kann) und asymptomatischem Primarius (Darmtumor, der aktuell keine Beschwerden verursacht) eine Entfernung des Primarius (= Darmtumor) erfolgen?

Hinweis: Bei Personenbezeichnungen und personenbezogenen Hauptwörtern wurde die männliche Form im Sinne der Gleichbehandlung grundsätzlich für alle Geschlechter verwendet und beinhaltet daher keine Bewertung.

Wir bitten Sie nun noch um weitere Informationen zu Ihrer Person:

Welche der folgenden Beschreibungen trifft auf Sie zu (ggf. mehrere Angaben):

☐ Ich bin Patient/in mit Darmkrebs (kolorektales Karzinom)

☐ Ich bin Angehörige/r eines/r Patienten/in mit Darmkrebs (kolorektales Karzinom)

☐ Ich bin beruflich in der Versorgung von Patienten/innen mit Darmkrebs (kolorektales Karzinom) tätig,

☐ Möchte ich nicht beantworten

☐ Anderes, nämlich:____

<u>Für Patienten und Angehörige</u>

Wir hätten gerne folgende Informationen zur erkrankten Person:

1. Alter der erkrankten Person

☐	Unter 65 Jahre	☐	65 bis 80 Jahre	☐	über 80 Jahre
☐	möchte ich nicht beantworten				

2. Geschlecht der erkrankten Person

☐ weiblich ☐ männlich
☐ möchte ich nicht beantworten

3. In welchem Alter wurde der Darmkrebs bei der erkrankten Person festgestellt?

☐	Unter 65 Jahre	☐	65 bis 80 Jahre	☐	über 80 Jahre
☐	möchte ich nicht beantworten				

<u>Wenn Sie beruflich in der Versorgung von älteren Patienten/innen mit der Krebserkrankung des Dick- und Mastdarms (kolorektales Karzinom) tätig sind:</u>

Beruf:	
Ggf. Fachrichtung oder Zusatzbezeichnung angeben:	

Indem Sie an der Umfrage teilnehmen, willigen Sie ein, dass wir Ihre Fragen anonym veröffentlichen dürfen.

Sie können uns helfen, weitere Rückmeldungen auf unseren Fragebogen zu erhalten, wenn Sie den Link zur Online-Umfrage oder eine Kopie des Formulars an andere Personen weitergeben.

Wir bedanken uns für Ihre Teilnahme an diesem wichtigen Projekt!
