# Supplementary material for: Top 10 research priorities in colorectal cancer: results from the Colorectal Cancer Priority-Setting Partnership
Source: J Cancer Res Clin Oncol. 2022 May 17;149(4):1561–8. doi: 10.1007/s00432-022-04042-w (PMC10020251; doi:10.1007/s00432-022-04042-w)
Supplement: Supplementary file 4 — Supplement 4. Results of interim ranking [file 432_2022_4042_MOESM4_ESM.pptx]

## Slide 1
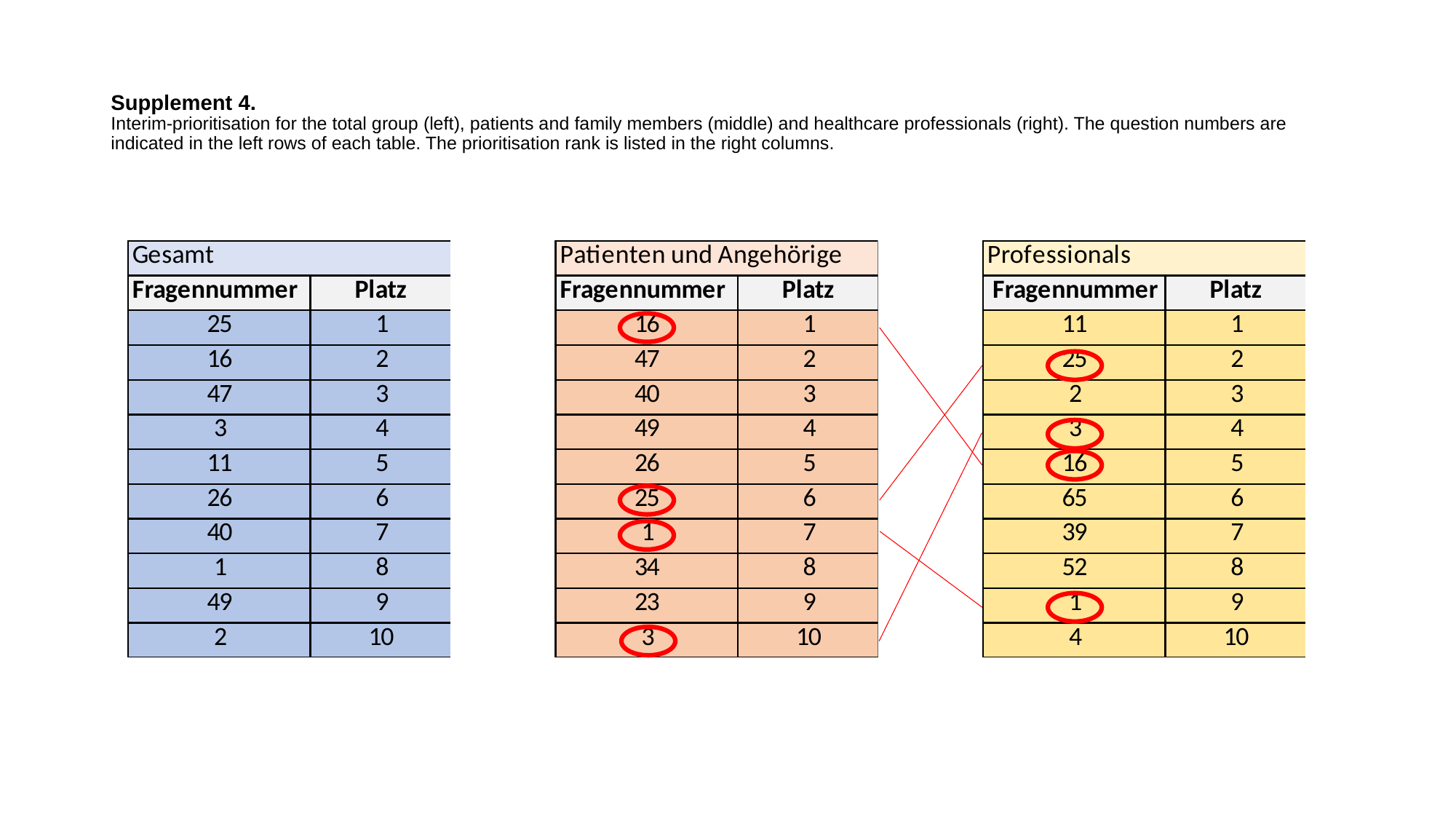

# Supplement 4. Interim-prioritisation for the total group (left), patients and family members (middle) and healthcare professionals (right). The question numbers are indicated in the left rows of each table. The prioritisation rank is listed in the right columns.
