# Supplementary material for: Top 10 research priorities in colorectal cancer: results from the Colorectal Cancer Priority-Setting Partnership
Source: J Cancer Res Clin Oncol. 2022 May 17;149(4):1561–8. doi: 10.1007/s00432-022-04042-w (PMC10020251; doi:10.1007/s00432-022-04042-w)
Supplement: Supplementary file 5 — Supplement 5. Research priorities 11–20 of the Colorectal Cancer Priority-Setting Partnership [file 432_2022_4042_MOESM5_ESM.docx]

**Supplement 5.** Research priorities 11-20 of the Colorectal Cancer Priority Setting Partnership

| **11** | What is the best way to treat liver metastases in colorectal cancer? |
| --- | --- |
| **12** | What is the best treatment for peritoneal carcinomatosis (tumor metastases in the peritoneum) in patients with colorectal cancer? |
| **13** | Is the quality of life of colorectal cancer patients with a stoma improved by care from stomatherapists, and how should such care be arranged (e.g., outpatient, inpatient, before surgery, duration of care from stomatherapists)? |
| **14** | How can the quality of life of colorectal cancer patients and their risk of recurrence be influenced during follow-up (e.g., nutritional counseling, sport, psychosocial factors)? |
| **15** | Depending on the tumor stage and risk profile, at what intervals and for how long should colorectal cancer continue to be followed up, and what techniques should be used (e.g., new biomarkers, liquid biopsy [= detection of tumor cells in blood], “personalized follow-up”)? |
| **16** | How can the patient’s treatment response and individual prognosis be predicted at the time of diagnosis of colorectal cancer (e.g., by means of biomarkers)? |
| **17** | How can the adverse effects and consequences of radiotherapy be prevented and treated in patients with colorectal cancer? |
| **18** | Who should perform the follow-up of patients with colorectal cancer (e.g., primary care physician, cancer centre, oncologist), and how can compliance (active cooperation by the patient) be improved (e.g., patient-reported outcomes measures [PROMs; = instruments for documentation of the results of treatment by the patients themselves], apps, follow-up passport)? |
| **19** | How can complications following surgery for colorectal cancer be reduced and treated? |
| **20** | What kinds of sport and exercise can colorectal cancer patients perform in what situation (e.g., after surgery, with or without a stoma, or during chemotherapy), how may this affect the outcome, e.g., with regard to hernia prevention, complication rate, and risk of recurrence, and what activities should be avoided? |
