# Supplementary material for: Top 10 research priorities in colorectal cancer: results from the Colorectal Cancer Priority-Setting Partnership
Source: J Cancer Res Clin Oncol. 2022 May 17;149(4):1561–8. doi: 10.1007/s00432-022-04042-w (PMC10020251; doi:10.1007/s00432-022-04042-w)
Supplement: Supplementary file 6 — Supplement 6. Top 1–20 research priorities in German [file 432_2022_4042_MOESM6_ESM.docx]

**Supplement 6.** Finale top 11-20 priorities of the PSP Colorectal Cancer (Forschungspartnerschaft Darmkrebs) in its German original.

| **1** | Wie radikal muss bei Darmkrebs je nach Tumorstadium operiert werden (z.B. in Bezug auf Beckenexenteration (= (radikale) operative Entfernung von mehreren Organen im Becken), Kontinenzerhaltung (Fähigkeit, Stuhl willkürlich zurückzuhalten), Lymphknotenentfernung)? |
| --- | --- |
| **2** | Welche Maßnahmen helfen bei Darmkrebs bei der Bewältigung der Erkrankung und der Therapienebenwirkungen und -folgen (z. B. Darmverschluss, Durchfälle, Entzündungen am After, Inkontinenz, parenterale Ernährung (= Ernährung über die Venen), sexuelle Probleme, Folgen der Stomarückverlagerung)? |
| **3** | Welche Möglichkeiten bietet die personalisierte Therapie (z. B. Antikörpergabe, „targeted therapy“ (= zielgerichtete Therapie mit neuartigen Medikamenten) oder eine Immuntherapie bei Darmkrebs? |
| **4** | Verbessert ambulantes und stationäres Fachpersonal (Darmzentrum, Ernährungsberatung, onkologische Fachpflegekraft, Pflegedienst, Stomatherapeut, etc.) in der Betreuung von Darmkrebspatienten das Outcome (=Ergebnis)? |
| **5** | Welche gezielte Vorbereitung (Prehabilitation) kann das Outcome (= Ergebnis) der geplanten Therapie (Operation, Bestrahlung, Chemotherapie etc.) bei Darmkrebs verbessern? |
| **6** | Welchen Stellenwert hat die Komplementärmedizin (z. B. Meditation, Naturheilverfahren, Osteopathie, traditionelle chinesische Medizin) ergänzend zur Schulmedizin (z. B. in Bezug auf Symptomlinderung und Überleben) bei Darmkrebs? |
| **7** | Wie kann bei Enddarmkrebs LARS (Low anterior resection syndrom = gestörte Defäkation nach Enddarmentfernung) effektiv verhindert (z. B. mittels Rekonstruktionstechnik (= Technik der Wiederherstellung des Verdauungsweges, J-Pouch, transverse Kolosplastie, Seit-zu-End- Anastomose), pelvines Neuromonitoring (= Kontrolle der Nervenfunktion während der Operation)) oder behandelt werden (z. B. Biofeedback (= Erfassung und Darstellung bestimmter körpereigener Signale), Fasten, Irrigation (= Spülung des Darmes))? |
| **8** | Wie können bei Darmkrebs Nebenwirkungen der Chemotherapie (z.B. Polyneuropathie (= Schädigung von Nerven, die mit Missempfindungen und Schmerzen einhergeht), Übelkeit) vermieden und behandelt werden? |
| **9** | Was ist bei Darmkrebs die beste Reihenfolge der unterschiedlichen Therapiemöglichkeiten (Chemotherapie, Operation, Strahlentherapie) abhängig vom Tumorstadium? |
| **10** | Welche Maßnahmen können die Lebensqualität und den Allgemeinzustand bei Darmkrebs verbessern (z.B. Ernährung, Gespräche, Kultur, psychosoziale Unterstützung)? |
| **11** | Was ist bei Darmkrebs abhängig vom Befund (z. B. bezüglich Tumorstadium, Zeitpunkt des Auftretens und Anzahl der Metastasierung) die beste Therapie von Lebermetastasen (z.B. Cyberknife (robotergestützter Linearbeschleuniger für Strahlenchirurgie), Irreversible Elektroporation (= Weichgewebeablationsverfahren), Mikrowellenablation (= Zerstörung des Tumors durch Hitze von innen), Chemotherapie, Operation, Radiofrequenzablation (= Zerstörung des Tumors durch Zuführung von Hitze mit Hochfrequenzstrom), TACE/transarterielle Chemoembolisation (= gezielte Verstopfung von Arterien durch Chemotherapie))? |
| **12** | Was ist bei Darmkrebs die beste Therapie bei Peritonealkarzinose (= Tumorstreuung ins Bauchfell)? |
| **13** | Wird bei Darmkrebs das Leben nach Stomaanlage durch eine Betreuung durch Stomatherapeuten verbessert und wie sollen die Rahmenbedingungen beschaffen sein (z.B. ambulant, stationär, vor der Operation, Dauer der Betreuung)? |
| **14** | Wie können bei Darmkrebs Lebensqualität und Rezidivrisiko während der Nachsorge beeinflusst werden (z.B. Ernährung, Sport, psychosoziale Faktoren)? |
| **15** | In welchen Intervallen und wie lange sollte je nach Tumorstadium und Risikoprofil die Nachsorge bei Darmkrebs auch über die Heilungsbewährungsfrist von 5 Jahren hinaus durchgeführt werden und welche Untersuchungen (z. B. auch neue Biomarker, "liquid biopsy" (= Nachweis von Tumorzellen aus Blut), "maßgeschneiderte Nachsorge") sollte sie beinhalten, auch für die Therapiefolgen? |
| **16** | Wie kann man bei Darmkrebs bei Diagnosestellung das Therapieansprechen und die individuelle Prognose vorhersagen (z. B. durch Tumormarker)? |
| **17** | Wie können bei Darmkrebs Nebenwirkungen und Folgen der Strahlentherapie vermieden und behandelt werden? |
| **18** | Durch wen sollte bei Darmkrebs die Nachsorge durchgeführt werden (z.B. Hausarzt, Nachsorgezentren, Onkologe) und wie kann die Compliance (= aktive Mitwirkung der Patienten) verbessert werden (z. B. PROMs = patient reported outcomes measures = Instrumente zur Patientenselbstdokumentation des Behandlungsergebnisses, Apps, Nachsorgepass)? |
| **19** | Wie können Komplikationen (z. B. Nahtundichtigkeiten, Wundheilungsstörungen) nach der Operation des Darmkrebses vermieden, reduziert und behandelt werden? |
| **20** | Welche Sport- und Bewegungsarten sind bei Darmkrebs in welcher Situation (z.B. nach der Operation ggf. mit Stomaanlage oder während der Chemotherapie) möglich, wie können sie das Outcome (Ergebnis) z. B. in Bezug auf Hernienprophylaxe (= Vorbeugung von Bauchwandbrüchen), Komplikationsrate und Rezidivrisiko beeinflussen und welche sollten vermieden werden? |
