# Supplementary material for: Top 10 research priorities in colorectal cancer: results from the Colorectal Cancer Priority-Setting Partnership
Source: J Cancer Res Clin Oncol. 2022 May 17;149(4):1561–8. doi: 10.1007/s00432-022-04042-w (PMC10020251; doi:10.1007/s00432-022-04042-w)
Supplement: Supplementary file 7 — Supplement 7. List of the guidelines screened [file 432_2022_4042_MOESM7_ESM.docx]

**Supplement 7**

List of screened guidelines (October 2020):

(1) *S3-Leitlinie Kolorektales Karzinom* (Germany)

(2) Colorectal Cancer guidelines of the *National Institute for Health and Care Excellence* (NICE; UK)

(3) Colon Cancer guideline of the *National Comprehensive Cancer Network* (NCCN; USA)

(4) Guideline for metastatic and localized colorectal cancer of the *European Society for Medical Oncology* (ESMO).
